# Supplementary material for: From sequence to enzyme mechanism using multi-label machine learning
Source: BMC Bioinformatics. 2014 May 19;15:150. doi: 10.1186/1471-2105-15-150 (PMC4229970; doi:10.1186/1471-2105-15-150)
Supplement: Additional file 2 — Java code of ml2db. Additional file ml2db_code.tar.gz contains the Java source code to run the multi-label machine learning experiments and save the results to database. The code’s Javadoc is included. [file 1471-2105-15-150-S2.zip › additional file 2/ml2db/ml2db/doc/test/ml2db/mulan/attributesfilter/ArffGeneratorForFilterTests.html]

ArffGeneratorForFilterTests


JavaScript is disabled on your browser.


- Overview
- Package
- Class
- Use
- Tree
- Deprecated
- Index
- Help

- Prev Class
- Next Class

- Frames
- No Frames

- All Classes

- Summary:
- Nested |
- Field |
- Constr |
- Method

- Detail:
- Field |
- Constr |
- Method


test.ml2db.mulan.attributesfilter

## Class ArffGeneratorForFilterTests

- java.lang.Object
- - test.ml2db.mulan.attributesfilter.ArffGeneratorForFilterTests

- ---

    

  ```
  public class ArffGeneratorForFilterTests
  extends java.lang.Object
  ```

- - ### Constructor Summary

    Constructors

    Constructor and Description

    `ArffGeneratorForFilterTests()`
  - ### Method Summary

    Methods

    | Modifier and Type | Method and Description |
    | --- | --- |
    | `int` | `generateArff(java.lang.String dataProps)` Generate an arff file and return the arff record id |
    | `int` | `generateTestArff()` Generate the old test arff and return the arff record id |
    | `int` | `generateTrainArff()` Generate the old train arff and return the arff record id |

    - ### Methods inherited from class java.lang.Object

      `equals, getClass, hashCode, notify, notifyAll, toString, wait, wait, wait`

- - ### Constructor Detail


    - #### ArffGeneratorForFilterTests

      ```
      public ArffGeneratorForFilterTests()
      ```
  - ### Method Detail


    - #### generateArff

      ```
      public int generateArff(java.lang.String dataProps)
      ```

      Generate an arff file and return the arff record id

      Returns:


    - #### generateTestArff

      ```
      public int generateTestArff()
      ```

      Generate the old test arff and return the arff record id

      Returns:


    - #### generateTrainArff

      ```
      public int generateTrainArff()
      ```

      Generate the old train arff and return the arff record id

      Returns:


- Overview
- Package
- Class
- Use
- Tree
- Deprecated
- Index
- Help

- Prev Class
- Next Class

- Frames
- No Frames

- All Classes

- Summary:
- Nested |
- Field |
- Constr |
- Method

- Detail:
- Field |
- Constr |
- Method
